# Successful outcome of combined surgical and negative pressure therapy in complex enterocutaneous fistulas: a case report

**DOI:** 10.1093/jscr/rjad161

**Published:** 2023-03-31

**Authors:** Paola Solis-Pazmino, Leonardo Oliveira da Silva, Juan Quezada Huiracocha, Luis Henrique Saldanha, Augusta Avila, Guilherme Ribeiro, Rodrigo Pozza Pinto

**Affiliations:** Surgery Department, Hospital Dom João Becker, Santa Casa de Misericórdia in Porto Alegre (SCMPA), Porto Alegre, Brazil; Surgery Department, Hospital Dom João Becker, Santa Casa de Misericórdia in Porto Alegre (SCMPA), Porto Alegre, Brazil; Surgery Department, Hospital Dom João Becker, Santa Casa de Misericórdia in Porto Alegre (SCMPA), Porto Alegre, Brazil; Surgery Department, Hospital Dom João Becker, Santa Casa de Misericórdia in Porto Alegre (SCMPA), Porto Alegre, Brazil; Surgery Department, Hospital Dom João Becker, Santa Casa de Misericórdia in Porto Alegre (SCMPA), Porto Alegre, Brazil; Surgery Department, Hospital Dom João Becker, Santa Casa de Misericórdia in Porto Alegre (SCMPA), Porto Alegre, Brazil; Surgery Department, Hospital Dom João Becker, Santa Casa de Misericórdia in Porto Alegre (SCMPA), Porto Alegre, Brazil

## Abstract

An enterocutaneous fistula (ECF) is a complex medical issue that occurs when abnormal communication between the small intestine and the skin occurs. This can lead to the leakage of digestive contents, such as feces and food, onto the skin’s surface. The case of an 86-year-old woman is presented, who developed high-output ECF after undergoing Hartmann surgery, intestinal transit reconstruction for perforated diverticulitis and incisional hernia treatment involving hernioplasty and polypropylene mesh. The patient had suffered from a serous-purulent discharge from a low-volume surgical wound for several years. Despite optimizing the patient’s nutritional status, a laparotomy and small bowel resection were performed successfully. However, using vacuum dressing as a cover for the fistula in the lower gastrointestinal tract remains a subject of debate and limited research. No officially recognized international guidelines recommend its use for small bowel ECF.

## INTRODUCTION

An enterocutaneous fistula (ECF) is a complex medical condition characterized by an abnormal connection between the small intestine and the skin surface. This can cause digestive contents, such as food and feces, to leak out of the intestine and onto the skin [1]. The causes of ECFs can vary, including Crohn’s disease, cancer, injury or surgical complications. The condition can lead to severe complications such as infections, dehydration, malnutrition and even death [2].

Treating ECFs involves addressing the underlying cause, controlling symptoms and preventing complications. Both conservative and invasive management approaches are used, depending on the patient’s condition [3]. About one-third of ECFs will close spontaneously with traditional measures, particularly those with low output, long distances and located in the proximal small intestine. However, distal obstruction, epithelialized and short fistulous tract, infection and malignancy can prevent fistula closure.

In such cases, alternative therapies, such as negative pressure therapy, can isolate the wound from intestinal material and promote healing [2]. This involves combining total parenteral nutrition (TPN) with a device that separates the intestinal ends and allows the application of negative pressure without aspiration of the intestinal content. The goal is to establish a “floating stoma” that TPN can surround to isolate the wound from continuous contamination caused by enteric material spillage [4].

## CASE REPORT

An 81-year-old woman with a history of Hartmann surgery and intestinal transit reconstruction for perforated diverticulitis (15 years ago) and incisional hernia treated with hernioplasty and polypropylene mesh (10 years ago). Since then, the patient has had a serous-purulent discharge from an orifice in a low-volume surgical wound (SW).

**Figure 1:**
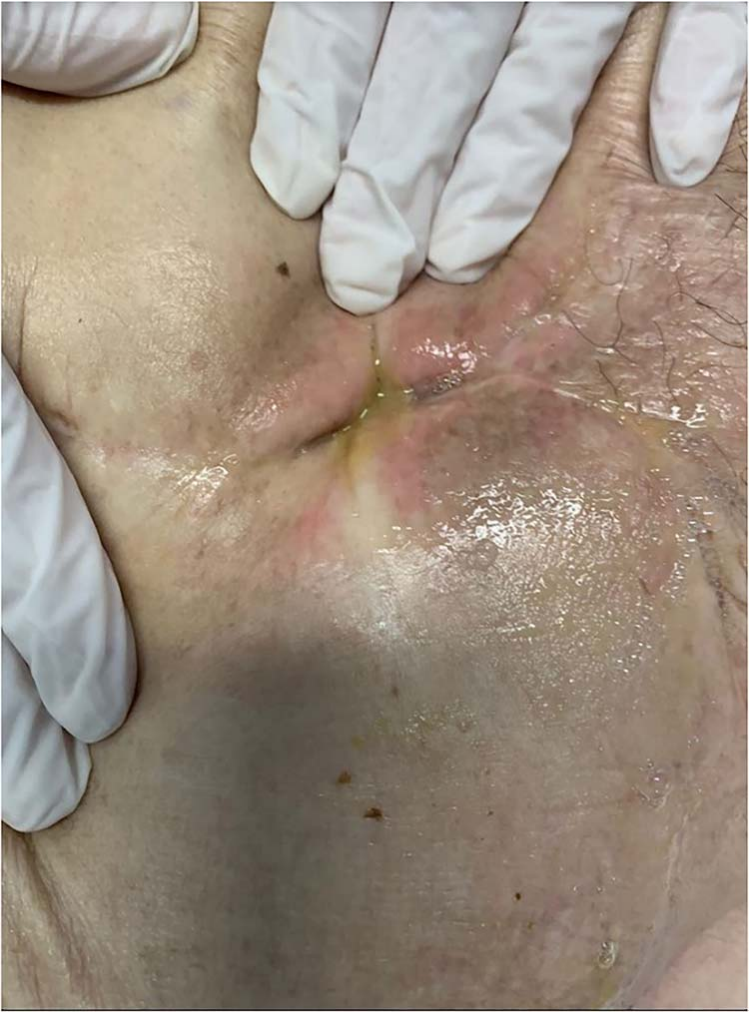
The surgical wound showed a fetid odor and color changes.

She was admitted to the digestive surgery service due to abdominal pain and fever. Upon physical examination, the SW showed a fetid odor, color and appearance change (Fig. 1).

**Figure 2:**
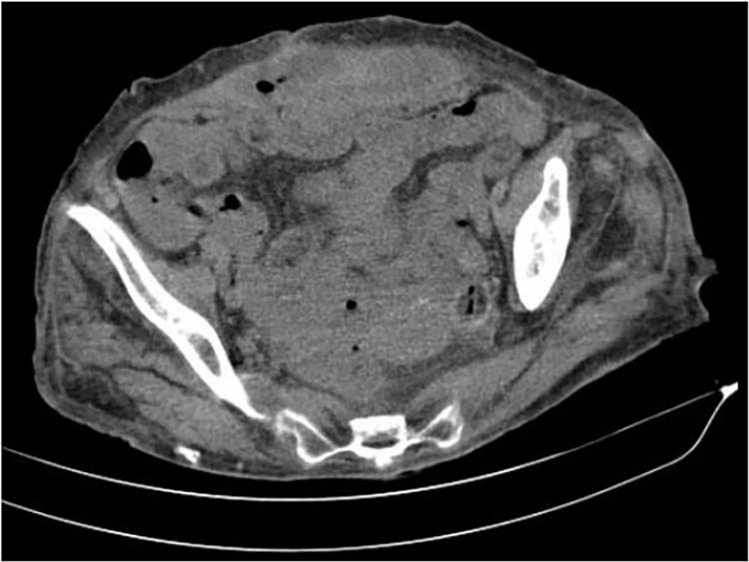
The axial CT section of the abdomen showing an abscess in the abdominal wall adjacent measured 12 × 4.0 × 13.0 cm.

The abdominal computed tomography (CT) scan showed an abscess in the abdominal wall adjacent to the previous mesh, measured 12 × 4.0 × 13.0 cm (Fig. 2). The patient’s laboratory test results showed a mild elevation of protein C-reactive (70.9 mg/L) with normal levels of white blood cells. The patient’s renal function and electrolyte levels were within normal limits.

## TREATMENT

**Figure 3:**
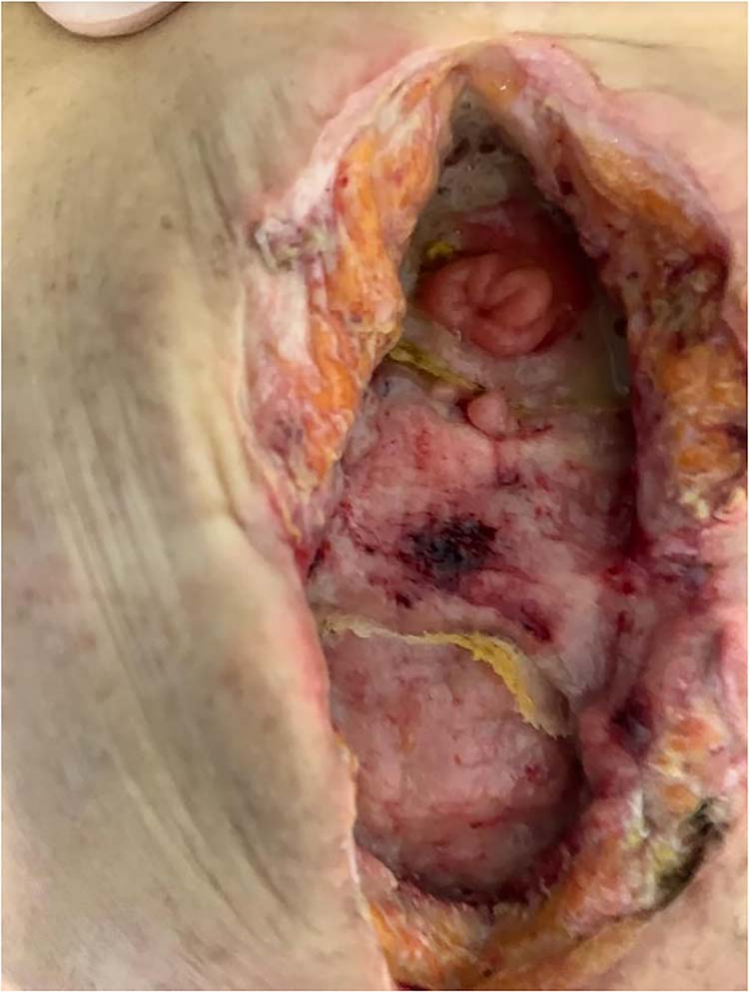
Entero-atmospheric labial fistula.

The patient was given antibiotic therapy (ampicillin + sulbactam) and underwent a procedure to drain a large abscess and remove mesh debris. This revealed an entero-atmospheric labial fistula (Fig. 3). Due to its severity and limited treatment options, it was decided to perform surgery to transform the fistula into a stoma by bringing the skin and subcutaneous tissue closer together.

However, the drainage and extravasation persisted and were complicated by chemical dermatitis. Another surgical intervention was performed with an aspiration probe positioned externally to reduce secretion and minimize dermatitis effects, but with limited success.

**Figure 4:**
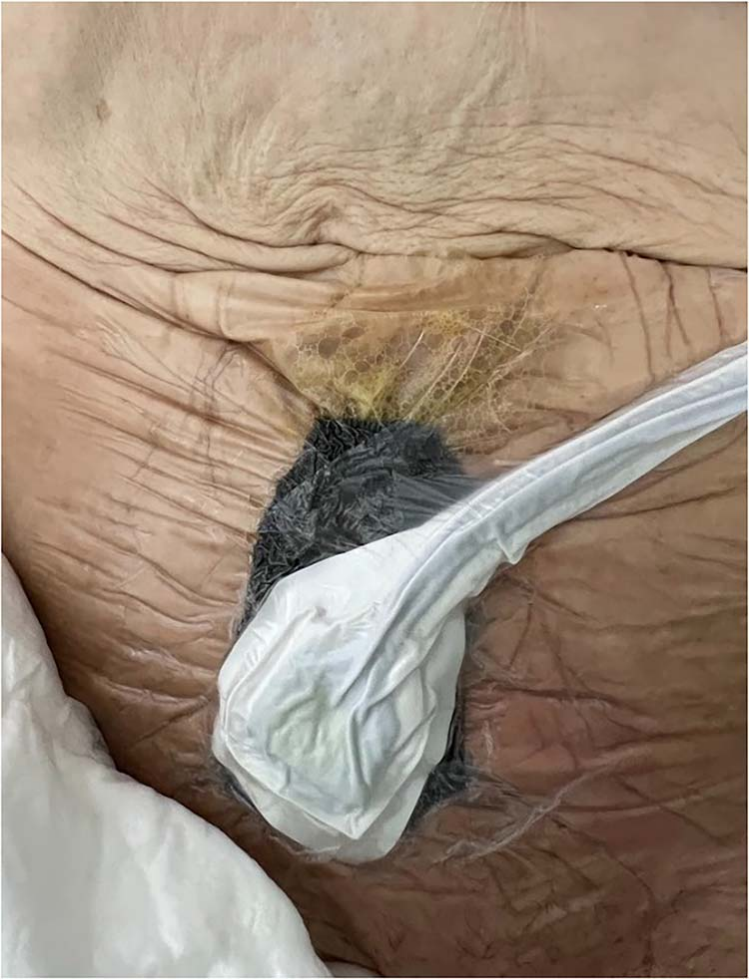
A vacuum dressing was then placed with a pressure of 70 mmHg and later changed to 100 mmHg.

**Figure 5:**
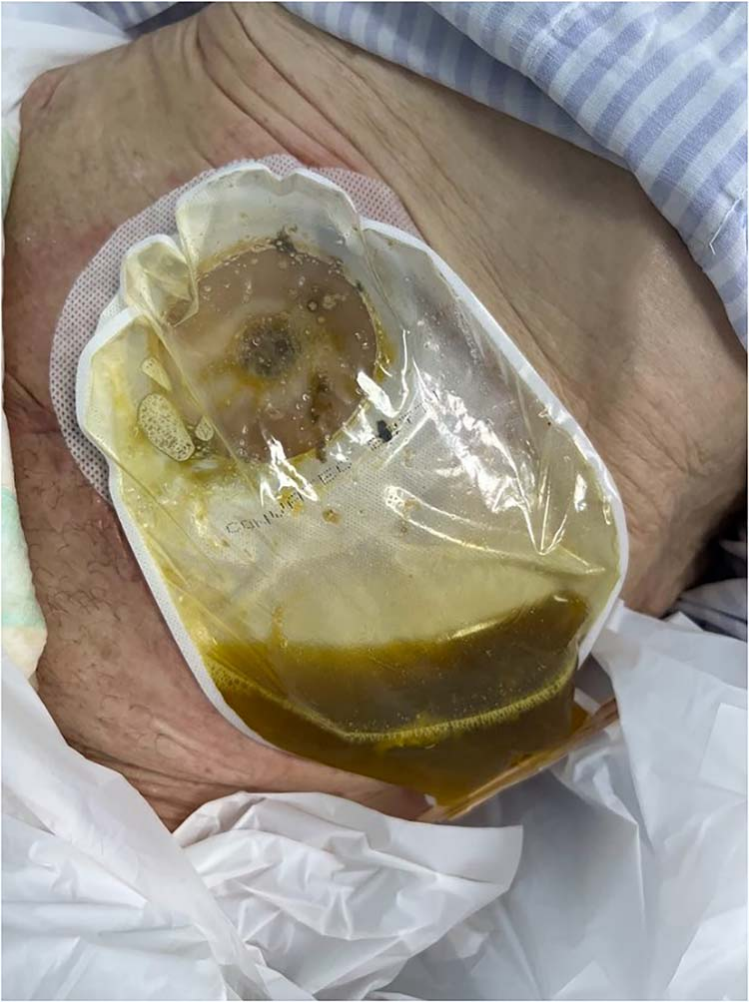
A karaya bag was placed and guided by vacuum therapy and stomahesive for skin protection.

**Figure 6:**
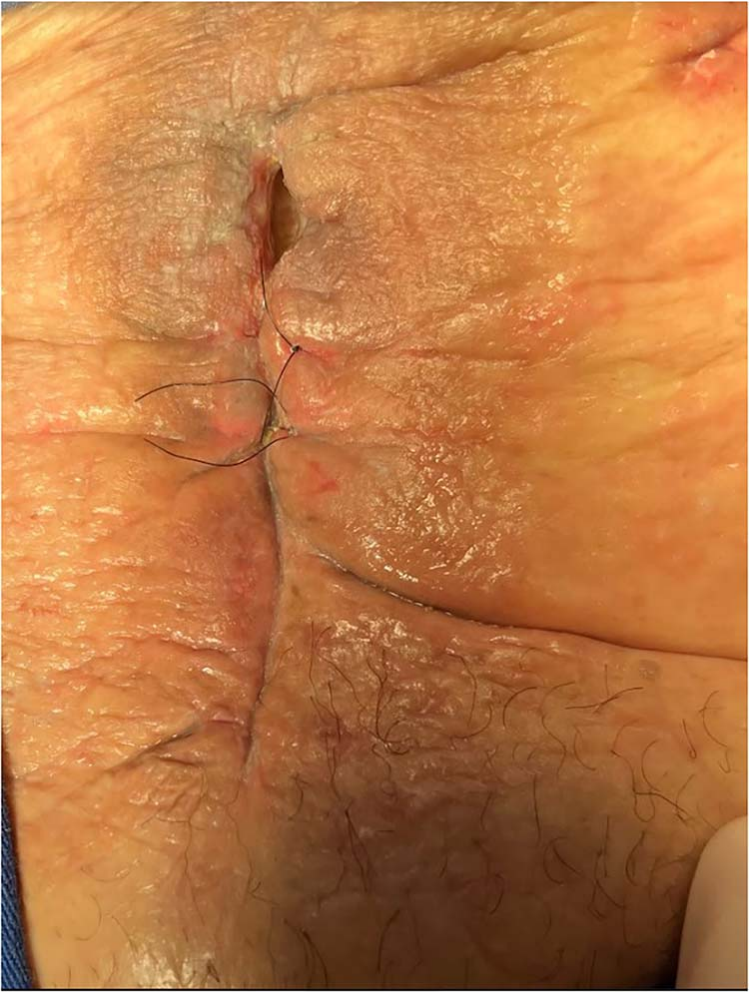
The patient had reduced drainage and an oriented fistula without chemical dermatitis.

**Figure 7:**
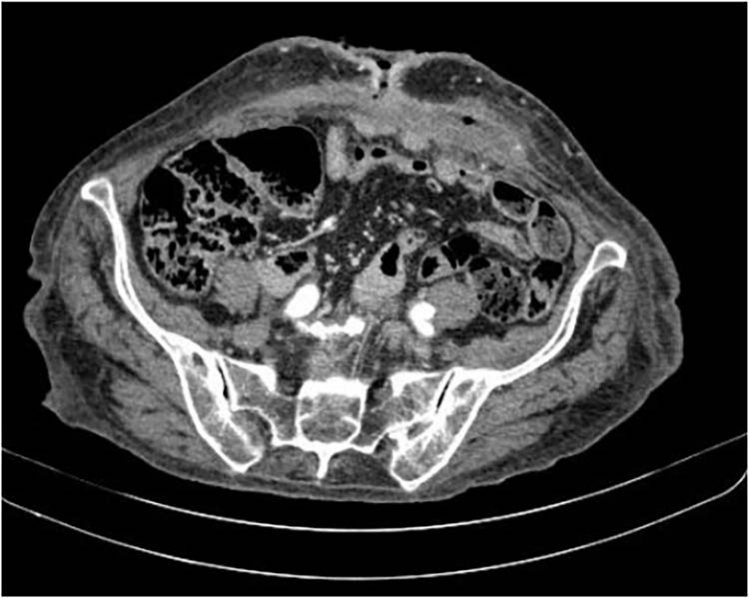
The axial CT section of the abdomen shows the loops in contact with the anterior abdominal wall at the level of the scar umbilical cord. The collection decreased compared with the previous CT scan in the subcutaneous tissue of the umbilical region.

A vacuum dressing was then placed with a pressure of 70 mmHg and later changed to 100 mmHg (Fig. 4). After 3 weeks, the sauce saturated and was removed. A karaya bag was placed and guided by vacuum therapy and stomahesive for skin protection (Fig. 5). The patient was started on a soft diet, gradually reduced parenteral nutrition, and a bland diet was initiated. The patient had reduced fistula drainage and was discharged with a healed SW, no chemical dermatitis and an oriented fistula (Figs. 6 and 7).

## DISCUSSION

ECFs can be classified based on the location and size of the fistula and the type of contents leaking. For example, a sizeable ECF that allows large amounts of feces to leak out may be classified as a high-output fistula [2]. We describe the case of an older woman who experienced a fistula recurrence, which required complex management before ultimately achieving closure using a vacuum dressing.

Negative pressure wound therapy (NPWT) is a type of wound care treatment that uses suction to promote wound healing [5]. This treatment method has gained popularity recently, with vacuum-assisted closure (VAC) devices becoming increasingly common [6]. NPWT works by applying a controlled amount of negative pressure to the wound site, which helps to remove excess fluid and promote the flow of oxygen and nutrients to the wound bed. This, in turn, helps to encourage new tissue growth and support the healing process. In addition, the negative pressure generated by the device helps to protect the skin and reduce the risk of wound dehiscence, which can occur when the wound reopens after initial closure.

The use of VAC devices in wound care has been shown to have several benefits, including faster wound healing times, reduced risk of infection and improved wound closure, according to the available literature (most of them are case reports) [7–12]. A systematic review included 151 patients who reported that the median closure rate with VAC was 64.6% (7.7–100%), with healing occurring within 58 (12–90) days [13]. In our patient, the mean duration of treatment was 12 days without the need for additional treatments.

However, some concerns regarding using VAC devices in wound care have been raised. Some healthcare professionals have raised the concern that VAC therapy can cause recurrent ECFs and wounds in the abdominal cavity [14]. In addition, there have been reports of increased mortality rates among patients undergoing VAC therapy [15], although this remains a matter of debate among healthcare professionals.

## CONCLUSION

NPWT using VAC devices is valuable in managing complex wounds. Although the benefits of this treatment are clear, it is essential for healthcare professionals to consider the potential risks associated with its use and to monitor patients carefully for any adverse effects. Overall, NPWT is a promising development in the field of wound care and has the potential to improve patient outcomes significantly.
